# The Spatial Properties of L- and M-Cone Inputs to Electroretinograms That Reflect Different Types of Post-Receptoral Processing

**DOI:** 10.1371/journal.pone.0121218

**Published:** 2015-03-18

**Authors:** Mellina M. Jacob, Gobinda Pangeni, Bruno D. Gomes, Givago S. Souza, Manoel da Silva Filho, Luiz Carlos L. Silveira, John Maguire, Neil R. A. Parry, Declan J. McKeefry, Jan Kremers

**Affiliations:** 1 Department of Ophthalmology, University Hospital Erlangen, Erlangen, Germany; 2 Instituto de Ciências Biológicas, Universidade Federal do Pará, Belém, Pará, Brazil; 3 Núcleo de Medicina Tropical, Universidade Federal do Pará, Belém, Pará, Brazil; 4 Centre for Hearing and Vision Research, Institute of Human Development, University of Manchester, United Kingdom; 5 Vision Science Centre, Manchester Royal Eye Hospital, Central Manchester University Hospitals NHS Foundation Trust, Manchester Academic Health Science Centre, Manchester, United Kingdom; 6 School of Optometry and Vision Science, University of Bradford, United Kingdom; University Zürich, SWITZERLAND

## Abstract

We studied the spatial arrangement of L- and M-cone driven electroretinograms (ERGs) reflecting the activity of magno- and parvocellular pathways. L- and M-cone isolating sine wave stimuli were created with a four primary LED stimulator using triple silent substitution paradigms. Temporal frequencies were 8 and 12 Hz, to reflect cone opponent activity, and 30, 36 and 48 Hz to reflect luminance activity. The responses were measured for full-field stimuli and for different circular and annular stimuli. The ERG data confirm the presence of two different mechanisms at intermediate and high temporal frequencies. The responses measured at high temporal frequencies strongly depended upon spatial stimulus configuration. In the full-field conditions, the L-cone driven responses were substantially larger than the full-field M-cone driven responses and also than the L-cone driven responses with smaller stimuli. The M-cone driven responses at full-field and with 70° diameter stimuli displayed similar amplitudes. The L- and M-cone driven responses measured at 8 and 12 Hz were of similar amplitude and approximately in counter-phase. The amplitudes were constant for most stimulus configurations. The results indicate that, when the ERG reflects luminance activity, it is positively correlated with stimulus size. Beyond 35° retinal eccentricity, the retina mainly contains L-cones. Small stimuli are sufficient to obtain maximal ERGs at low temporal frequencies where the ERGs are also sensitive to cone-opponent processing.

## Introduction

There is increasing evidence that flicker electroretinograms (ERGs) to stimuli containing luminance and red-green chromatic information are governed by two mechanisms that probably reflect the activity of the magno- and parvocellular retino-geniculate-cortical pathways [1–3]. At high temporal frequencies (> 30 Hz), the ERG has properties that resemble those of the magnocellular-based luminance pathway. At intermediate frequencies (8–12 Hz), the ERG has properties that are reminiscent of those of the red-green chromatic channel based on activity of the parvocellular pathway.

L- and M-cone isolating stimuli contain luminance and red-green chromatic information and thus can be used to study cone input to the two post-receptoral pathways. For instance, the relative strengths of signals driven by the long (L-) and middle (M-) wavelength sensitive cones in the two channels can be estimated [4,5]. It was found that the L- to M-cone response ratio is variable between individuals but generally larger than unity in ERGs at high temporal frequencies, corresponding to psychophysical findings relating to the luminance channel [5]. This L/M ratio is further correlated with the ratio of L- to M-cone numbers [6] and of L- to M-cone photopigment density [5]. In contrast, the L/M response ratio is close to unity in the red-green chromatic channel and in the 12 Hz flicker ERG.

Isolation of the responses of single photoreceptor types can be achieved by the silent substitution technique [7–9]. The amount of hyperpolarization of the photoreceptors after a photoisomerization is always the same and does not depend on the wavelength of the absorbed photon (this is the so-called principle of univariance). As a result, a stimulus will not alter the excitation of a photoreceptor type (and thus is ‘silent’) when the number of photoisomarizations does not change. The number of isomerizations can be calculated if the fundamentals of the photoreceptors (their absorption spectra corrected for preretinal absorption) and the emission spectra of the primaries (light sources) in the stimulus are known. The number of photoreceptor types that can be stimulated independently by a stimulus equals the number of primaries with independent spectra.

The distribution of L- and M-cones has been studied using various techniques. Adaptive optics have revealed the pattern of L- and M-cone distribution in the central retina [10]. Gene expression, measured by cone pigment messenger RNA (mRNA), may give a reliable estimate of the L- and M-cone distribution also at larger eccentricities because M and L cones produce equal amount of mRNA. Thus, it is possible to analyze the differences in cone distribution between central and peripheral retina. It has been observed that the L-/M-mRNA ratio increases with increasing eccentricity [11–13].

ERG data can reveal similar eccentricity dependent changes in the ratios of L- to M-cone driven signals. Using custom-built equipment for wide-field multifocal ERG recordings with red and green LEDs as light sources, Kuchenbecker and colleagues [14], reported a large increase in the relative sensitivity to red light compared to green light in the far peripheral retina, suggesting an increase in L/M ratio with increasing eccentricity.

Challa and colleagues [15], used the double silent substitution method to investigate L- and M-cone driven ERG responses to flickering stimuli at two different temporal frequencies (12 and 30 Hz) and for different spatial stimulus configurations. Double silent substitution meant that, in addition to the cone type under examination, they could not fully control an additional S-cone contribution, although asserted that this would have a negligible impact. Their results showed that at 12 Hz the L/M cone ratio was about unity for all stimuli. At 30 Hz the L/M ratio strongly depended on stimulus size, implying that the relative input strengths of the L- and M-cones to the non-opponent mechanism change with retinal eccentricity [confirming earlier data [4]].

However, for complete isolation of the L- or M-cone responses, triple silent substitution is necessary (so that three photoreceptor types are silenced leaving only the fourth photoreceptor type-either the L- or the M-cones- to respond) which is only possible with stimulators having at least four independent primaries [16]. This assumes that responses of intrinsically responsive retinal ganglion cells that contain melanopsin do not contribute to the ERG. Full isolation could not be achieved in previous electrophysiological experiments using CRT screens [1,15] or two-primary LED stimulators [14]. In previous experiments, the triple silent substitution method was verified in dichromats, who lack either L- or M-cones [17–20]. Another disadvantage of the previous methods was that full field stimulation (stimulation of the complete retina) could not be achieved. It was therefore not possible to study the spatial arrangement of the L- and M-cone driven signals in the far periphery (beyond about 50°).

The aim of the present study was to examine in detail the spatial distribution of L- and M-cone driven responses in ERGs reflecting activity of the two post-receptoral (presumably magnocellular and parvocellular) pathways while using the triple silent substitution paradigm and including conditions wherein far peripheral cones were stimulated so L- and M-cone driven ERG signals can be studied for the complete retina. To be able to do this, we used a Ganzfeld bowl with four differently colored LED arrays as a stimulator.

The results confirm the presence of two separate mechanisms that mediate ERG responses. At high temporal frequencies (> 30 Hz), the response amplitude is positively correlated with stimulus area. At intermediate temporal frequencies (8 and 12 Hz) the responses are already maximal for a relatively small stimulus area.

## Materials and Methods

### Subjects

In total, 8 subjects participated in the experiments. Three experimental procedures were employed (see below). The main experiment (Experiment 1) was conducted with four subjects (three males and one female, aged between 28 and 53 years). In Experiment 2 one female subject (age 28 years) participated. In Experiment 3, five male observers, ranging in age between 29 and 54 years participated. All observers were healthy and underwent an ophthalmological evaluation to exclude the presence of any retinal disorders. All had normal color vision as established with the Farnsworth Munsell D-15 color arrangement test and the HMC anomaloscope (Oculus Optikgeräte GmbH, Wetzlar, Germany). All subjects signed an informed consent prior to the experiments, which followed the tenets of the Declaration of Helsinki. The protocol was approved by the local institutional ethics committee (medical faculty of the University Erlangen-Nürnberg) on human experimentation.

### Visual stimulation

**Fig 1:**
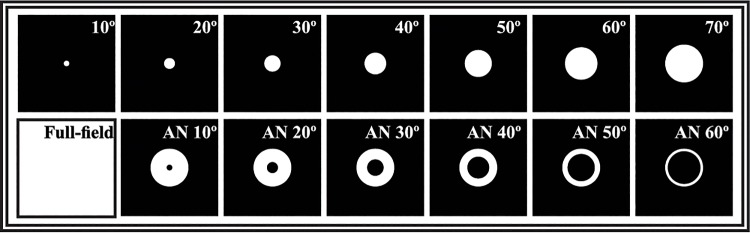
Spatial stimulus configuration of circular and annular stimuli used for ERG recordings.

The visual stimuli were generated by a RETIport system (Roland Consult Stasche & Finger GmbH, Brandenburg a. d. Havel, Germany) presented using a Ganzfeld bowl (Q450SC; Roland Consult), containing six arrays of differently colored light-emitting diodes (LEDs), which were independently driven by the RETIport system. For the current experiment, we used four narrow band LEDs: green, orange, blue, and red. Stimuli were sine-wave modulations of the luminance output of each of the LEDs. The responses were measured for 14 different spatial stimulus configurations: one full-field stimulus, seven circular stimuli varying in size between 10° and 70° in 10° steps, and six annular stimuli with 70° outer diameter and inner diameters between 10° and 60° varying in 10° steps (see Fig. 1). The spatial configurations were created by black cardboard field stops that were positioned 3 cm from the observer. Since the observers accommodated on a fixation spot at about 30 cm distance, the edges of the field stops were extremely blurred. The experiments were performed in a dark room. In Experiment 2, the effects of stray light were studied. In that experiment, we used white card board that was illuminated and the experiments were performed under normal room lighting.

In all experiments, L- and M- cone isolating sine-wave stimuli were created using triple silent substitution [7–9]. In Experiments 1 and 2, the mean luminance was 284 cd/m^2^. The mean chromaticity was white with CIE1931 coordinates: x = 0.3531, y = 0.3181. The luminance for each LED array was: 124 cd/m^2^ green (523 nm, CIE1931: x = 0.2016, y = 0.7371), 67 cd/m^2^ orange (594 nm, CIE1931: x = 0.5753, y = 0.4240), 44 cd/m^2^ blue (469 nm, CIE1931: x = 0.1255, y = 0.0926), and 49 cd/m^2^ red (638 nm, CIE1931: x = 0.6957, y = 0.2966). In Experiment 3, the mean luminance was also 284 cd/m² (red, orange, green, and blue LEDs: 80, 160, 40, and 4 cd/m^2^ respectively) but with a reddish mean chromaticity (CIE1931 coordinates: x = 0.5951, y = 0.3857).

In Experiments 1 and 2, the cone contrast was 10%. The contrast in the silenced photoreceptors was 0%, except for the M-cone isolating condition where S- cone contrast was-2% and rod contrast was 2% (the minus sign indicates a counter-phase modulation whereas a positive sign indicates in-phase modulation). We were forced to allow this small S- cone and rod modulation to ensure that a 10% M-cone modulation was possible. In previous experiments [17], we found that the response amplitudes of S-cone- and rod-driven responses were substantially smaller than M-cone driven responses even if the S- cone- and rod-contrasts were substantially higher than the M-cone contrast. The small rod and S-cone modulation in the “M-cone isolating” condition will therefore result in negligible S-cone- and rod-driven responses. In Experiment 3, pure L- and M-cone isolating conditions, each with 18% cone contrast and 0% contrast in the silenced photoreceptors, were used.

In Experiment 1, the measurements were performed at five temporal frequencies: 8, 12, 30, 36 and 48 Hz. In Experiment 2, we used 12 and 30 Hz stimuli. In Experiment 3, we used 12 and 36 Hz stimuli.

### ERG recording

Electroretinographic recordings were acquired using the RETIport system (Roland Consult). They were recorded monocularly from either the right or left eye. The pupils were dilated with a drop of 0.5% tropicamide (Pharma Stulln GmbH, Stulln, Germany). Anesthetic eye drops of oxybuprocain hydrochloride 0.04% (Dr. Mann Pharma, Berlin, Germany) were administered when requested by the observer. A corneal fiber (DTL) electrode, placed over the lower conjunctiva and attached close to the inner and outer cantus, was used as active electrode. Gold cup electrodes were placed on the forehead and the ipsilateral temple and used as ground and reference electrodes respectively. Gold cup electrodes were positioned with an electrode paste (DO Weaver & Company, Aurora, Colorado, USA), after the skin was cleaned with Nuprep abrasive skin preparing gel (DO Weaver & Company). Impedances were generally below 5 kΩ.

ERGs were averaged from between 80 and 160 epochs each lasting one second. To avoid onset artifacts, the recordings commenced 2 sec after start of the stimulus. The signals were amplified and band-pass filtered between 1 and 300 Hz and they were digitized at 1024 Hz.

### Analysis

The ERG responses were Fourier analyzed (using FFT) with custom written programs in MATLAB R2011b (Mathworks, Massachusetts, USA). Amplitudes (*amp(Fr)*) and phases of the first (fundamental) harmonic components were extracted. From the FFTs, the average of component amplitudes at the stimulus frequency +1 Hz and-1 Hz (*Amp (Fr+1)* and *Amp (Fr-1)*; e.g. with 12 Hz stimuli, these were the components at 11 Hz and 13 Hz) were calculated and defined as the noise in the concerning experiments. The signal-to-noise ratio SNR is given by:

SNR=Amp(Fr)/((Amp(Fr−1)+Amp(Fr+1))/2) (1)

Signals were accepted for further analysis when the SNR was larger than 2.

The Fourier analysis returns phases within a 360° range. Thus, the absolute phase can differ by multiples of 360°. To set the absolute phases of responses from different subjects we assumed that they did not differ more than 180°. Amplitudes and phases were averaged arithmetically.

## Results

### Experiment 1

**Fig 2:**
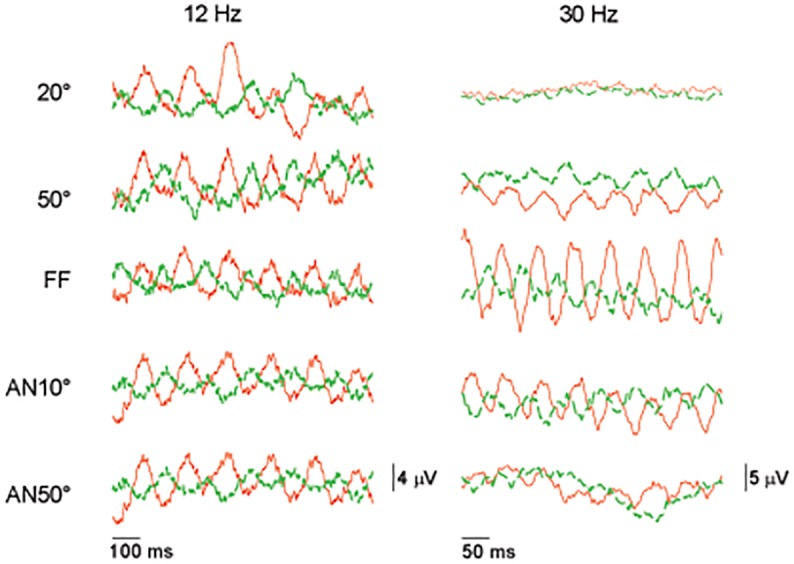
Original ERG traces. Original ERG traces for L- (10% cone contrast, dark red traces) and M- cone (10% cone contrast, green dashed traces) isolating stimuli, measured in one subject. The responses from two temporal frequencies (500 ms excerpts at 12 Hz and 200 ms excerpts at 36 Hz) and five different spatial configurations (20°, 50°, FF, AN10°, AN50°) are shown.

In Experiment 1, the L- and M-cone driven ERGs were measured at five temporal frequencies with the 14 spatial stimulus configurations (see Fig. 1), from four subjects. Original recordings, measured in one subject, obtained with 12 Hz and 30 Hz L- and M- cone isolating stimuli, each at 10% cone contrast, are shown in Fig. 2. The figure shows the responses for a representative set of five spatial stimulus configurations. The results of the responses to the remaining spatial configurations are not shown for clarity. It can be observed that the responses to 12 Hz stimuli (left column) do not change as strongly with spatial stimulus configuration as the 30 Hz responses (right column).

While the amplitude of the L- cone driven responses is larger than those of the M-cone driven responses at high temporal frequencies (and particularly for the full field [FF] responses), the amplitude differences are smaller at 12 Hz. At 8 Hz almost no amplitude differences were observed.

**Fig 3:**
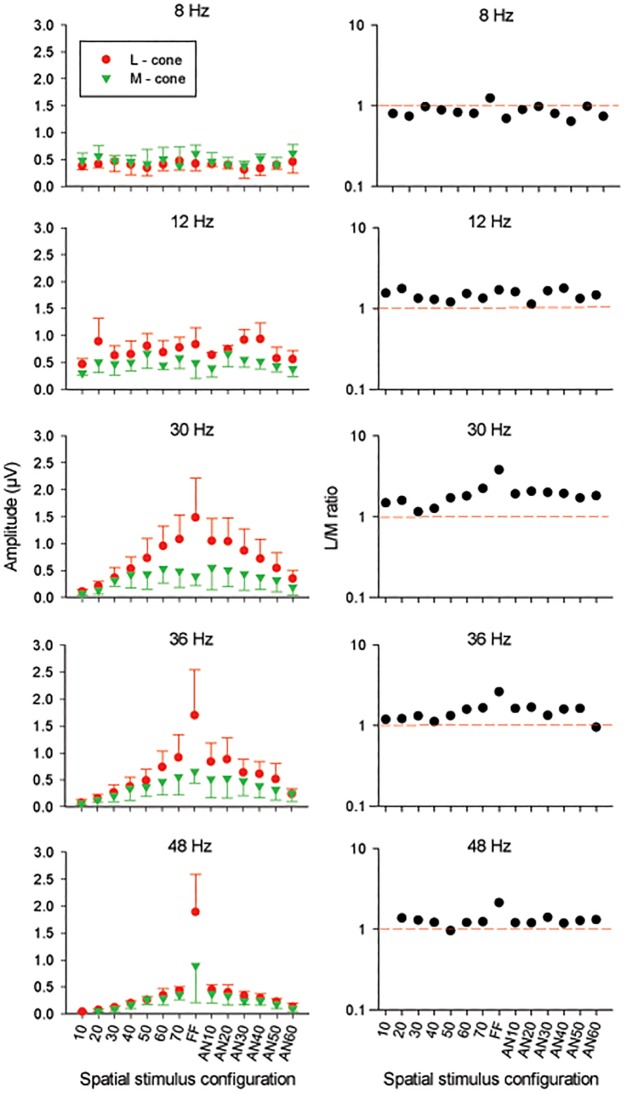
Amplitude data. Averaged amplitude (left plots; linear scale) for L- (red circles) and M- cone (green triangles) driven ERG responses as a function of stimulus configuration at 5 different temporal frequencies. The errors bars indicate the standard deviation. L-M ratio (right plots) estimated from the averaged amplitude at each temporal frequency. The red dashed lines indicate L/M ratio corresponding to unity.

The averaged amplitudes and L/M amplitude ratio data from the four subjects who participated in this experiment are displayed in Fig. 3 as a function of spatial stimulus configuration. The data are shown separately for each temporal frequency. The SNR varied between about 16 and 0.5 and depended on subject, temporal frequency and spatial configuration. The SNR was large for the 30 and 36 Hz stimuli and the FF conditions. The SNR for the 10°, 48 Hz M-cone isolating condition was below the criterion of 2 for all four subjects and these data therefore were not included.

At high temporal frequencies (30, 36 and 48 Hz), the response amplitudes increased with increasing stimulus area (i.e. with increasing diameter of the circular stimuli and decreasing diameter of the central ablation of the annular stimuli). The FF L-cone driven responses were considerably larger than those to the other stimuli. In contrast, the FF M-cone driven responses exhibited relatively smaller increases compared to those obtained with the 70 stimuli. The amplitude ratios decreased with increasing temporal frequency (Fig. 3; right plots) and were closer to unity for the 48 Hz stimuli, except for the FF responses. The L/M amplitude ratios decreased (and approached unity) when the ERGs were measured with smaller stimuli.

At 8 and 12 Hz, the averaged amplitudes of L- and M-cone driven responses were very similar and did not depend strongly on spatial stimulus configuration. As a result, the L/M ratios were close to unity for all stimulus configurations. In conclusion, the ERG amplitudes measured at 8 and 12 Hz, on the one hand, and at high temporal frequencies on the other, display distinctly different characteristics. This finding is in agreement with previous data [1–3,15] that demonstrate the ERGs at intermediate and high temporal frequencies are governed by two distinct mechanisms.

**Fig 4:**
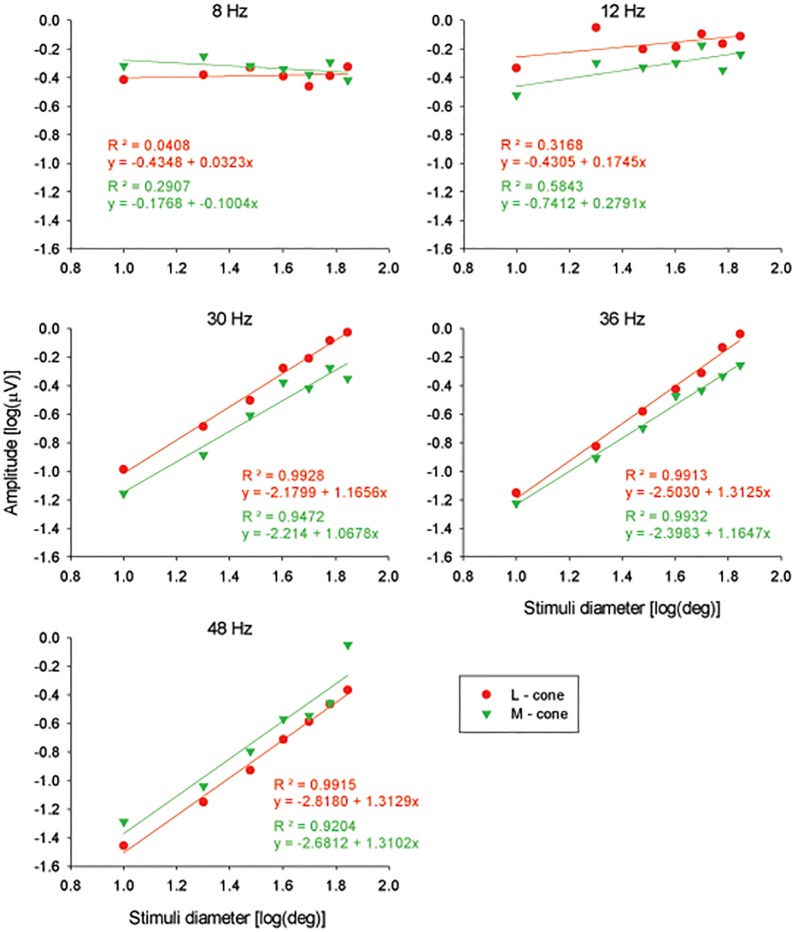
Amplitudes as a function of stimulus diameter. Linear regression analysis between the logarithm of amplitude and the logarithm of stimulus diameter for L-cone (red circles) and M-cone (green triangles) driven ERGs at 5 different temporal frequencies. The regression coefficient (R^2^) and the linear regressions are showed for each regression.

To study the dependency of amplitude on stimulus size in more detail, we plotted them as a function of the circular stimulus diameter (i.e. excluding the responses to FF and the annular stimuli; see Fig. 4) using double logarithmic scaling. At 8 and 12 Hz, the response amplitudes were relatively constant. This result is surprising because it suggests that only a small retinal area when stimulated was sufficient to elicit a maximal response. At high temporal frequencies (30 Hz and above), the L- and M-cone driven response amplitudes were positively correlated with stimulus size. The relationship between amplitude and stimulus size was similar, but not identical, for L- and M-cone driven responses and was approximately linear in the double logarithmic plot with r^2^ values exceeding 0.92. The slopes of the linear regression were between 1.1 and 1.6 log(μV)/log(deg).

**Fig 5:**
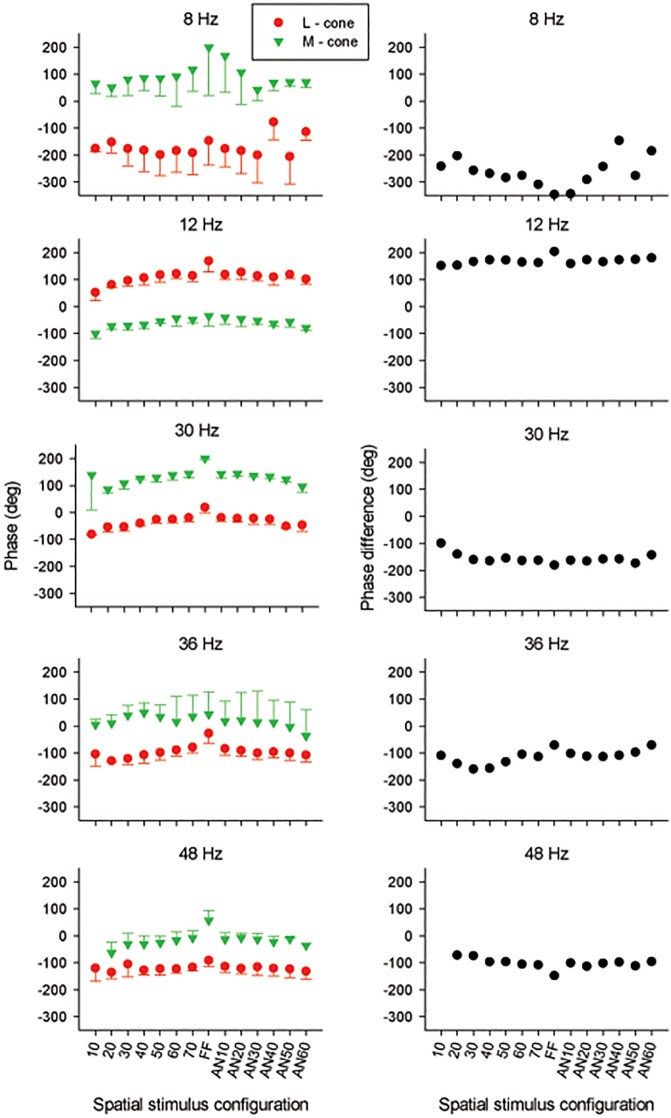
Phase data. Averaged phase values (left plots) for L- (red circles) and M- cone (green triangles) driven ERG responses as a function of stimuli configuration at 5 different temporal frequencies. The right plots correspond to the averaged L-M phase difference estimated from phase responses at each temporal frequency.

The averaged response phase obtained for each of the five temporal frequencies is shown as a function of spatial stimulus configuration in the left plots of Fig. 5. The responses were phase advanced when stimulus area increased for both circular and annular stimuli. The responses to full field stimuli were considerably phase advanced relative to the other responses. This was particularly the case at high temporal frequencies. Overall, the phases of L- and M-cone driven responses varied in a similar manner as a function of stimulus configuration. As a result, the averaged phase differences between the two (Fig. 5 right plots) were relatively independent of stimulus configuration. At 12 Hz, the phase difference was close to 180°, indicating that the L- and M-cones are operating in an opponent fashion. The phase differences were about 170° at 30 Hz stimuli and about 100° at 36 and 48 Hz.

### Experiment 2

The finding that the response amplitudes obtained at low temporal frequencies were largely independent of stimulus configuration (Figs. 3 and 4) was surprising. In a previous study [15], size dependent response amplitudes were found also at 12 Hz, even though the L/M ratios, measured at 12 and 30 Hz, were similar as in the present study. In the previous study, a CRT screen was used as stimulator and the non-stimulated area had the same mean luminance as the stimulus. In Experiment 1, the stimulus was blocked by black cardboard and the measurements were performed in a darkened room. Therefore the ERG responses obtained in Experiment 1 were possibly influenced by stray light. Although stray light would not be expected to have an effect only at low temporal frequencies, we repeated the 12 and 30 Hz measurements in one subject using illuminated white cardboard as field stops to suppress stray light.

The comparison between amplitude values from both experiments showed that amplitudes and phases obtained in the two experiments depended in a similar manner on spatial stimulus configuration (S1 Fig. and S2 Fig). The amplitudes acquired with the white surround were only slightly smaller than those obtained with a black surround. This was observed for both temporal frequencies. In conclusion, stray light probably had an influence on absolute response amplitudes but not on the relationship between amplitude and spatial stimulus configuration.

### Experiment 3

We also studied the effects of mean chromaticity because it was previously found that state of adaptation may strongly influence the results [21]. We therefore repeated the experiments using the same mean luminance but a different mean chromaticity. This allowed us to use larger stimulus strengths (18% cone contrast for L- and M-cone isolating stimuli). With the exception of one subject, the subjects that participated in this experiment did not participate in the main experiment. Although the absolute amplitudes were larger with the reddish background (probably caused by the larger cone contrast) the dependency of amplitude on stimulus configuration was very similar with the two backgrounds. The response phases also were very similar with the two backgrounds (S3 Fig. and S4 Fig.).

## Discussion

### Two separate post-receptoral mechanisms mediate ERG responses

In this study, triple silent substitution stimulation was used to investigate the spatial properties of L- and M-cone driven ERGs responses. The results indicate that, in agreement with previous data [1–3], two different mechanisms govern the ERG response. At high temporal frequencies (> 30 Hz), the response amplitude was positively correlated with stimulus area. The L/M response amplitude ratio was generally larger than one and increases with increasing stimuli size. In contrast, at intermediate temporal frequencies (8 and 12 Hz), the amplitudes were nearly constant for all stimulus configurations used in the present experiment and the L/M ratios were constant at about unity.

Previous data suggested that the ERGs at high temporal frequencies reflect activity of the luminance pathway. This is also in agreement with the notion that ERG and psychophysical measurements performed at high temporal frequency using heterochromatic flicker photometry results in nearly identical spectral sensitivities (the luminous efficiency function; V) with similar L/M ratios and with similar inter-individual variability [22–25]. The present data confirm that the average L/M ratio for spatially restricted stimuli is about two. It was proposed that the ERGs measured at 8 and 12 Hz reflect activity of the L-M cone opponent pathway. L-M cone opponent retinal ganglion cells can respond strongly to high temporal frequency luminance stimuli particularly in the far periphery [26]. However, the L/M ratio in in retinal ganglion cells bearing L-M cone opponent signals is about unity [27]. A ratio of about unity was also found for psychophysical flicker detection thresholds provided the L-M- chromatic pathway mediated the detection [5,27]. Indeed we also found an L/M ratio close to unity in the present study. Furthermore, the phase difference of about 180° between L- and M-cone driven responses suggest cone opponent processing. However, at higher temporal frequencies, where a luminance reflecting mechanism is proposed, the phase difference is also large. In addition to the absolute phase difference, the apparent latencies of L- and M-cone driven signals should be taken into account. The apparent latency can be obtained from the slope of the phase vs temporal frequency function. Our sampling of temporal frequencies is too coarse to estimate the apparent latencies. In a previous study with FF stimuli it was found that the apparent latencies of L- and M-cone driven ERG signals are very small at intermediate temporal frequencies whereas there are substantial differences at high temporal frequencies [18] and may cause the large differences between L- and M-cone driven phases at high temporal frequencies.

The fundamental components of the ERG responses to full field sinusoidal luminance stimuli display a minimum for temporal frequencies around 10 Hz [28–31]. Possibly, this minimum in the luminance response provides the possibility to study L-M opponent signals in the ERG. The minimum has been explained by linear interaction between ON and OFF responses [31]. However, the minimum in the fundamental component in luminance responses is accompanied by a maximum in the second harmonic component that cannot be explained by a simple linear model. We therefore offered an alternative model on the basis of two independent ERG mechanisms [29]. It is unclear whether the two mechanisms in the luminance responses correspond to those in the responses to cone isolating stimuli.

### Dependency of ERG responses on spatial stimulus configuration

The amplitude data obtained at 8 and 12 Hz suggest that a relatively small stimulus is enough to elicit a maximal response. This was also found with a completely different ERG recordings setup with a different four primary LED stimulator [32]. The response amplitudes were smaller only for a 10° stimulus (Fig. 3). These results are in contrast with the data of a previous study [15], in which the amplitude varied as a function of the spatial configurations of the stimuli. The discrepancy cannot be explained by the effect of the stray light, as was shown by Experiment 2. Furthermore, stray light effects would also influence the high temporal frequencies responses. A possible explanation for the differences in the results between both studies is the use of different types of stimulators. Challa and colleagues [15] have measured ERGs in different spatial stimuli configuration, using a CRT screen as stimulator. As this type of stimulator is composed of three phosphors (red, green, and blue), the cone modulation is limited by a spectral overlap of these phosphors and by a restricted luminance. These limitations allow only a double silent substitution technique [33]. Furthermore, in contrast to stimuli provided with an LED stimulator, CRT stimuli are not purely sinusoidal and include a sudden rise of the excited phosphors followed by a decay phase and thus contain higher frequencies which may influence the ERG response. The key difference between the present study and that of Challa and colleagues [15] is in the behavior of the M-cone response. We show a constant relationship between L and M cone phase as stimulus size increases, whilst the CRT study exhibited constant L-cone phases but a change in M-cone phase. This was accompanied by a marked increase in L/M ratio with size and lends weight to the signal cancellation hypothesis, which we discuss below. In a parallel study we established this by comparing CRT and ganzfeld responses in the same subjects [32].

The response amplitudes obtained at high temporal frequencies depended on stimulus size. For circular stimuli with diameters between 10° and 70°, the L- and M-cone driven responses increased with increasing stimulus size in a similar manner (resulting in a constant L/M ratio) and could be described by an exponential function with an exponent of between 1.1 and 1.3 (Fig. 4). If the retina was completely homogeneous and every part of the retina would contribute equally to the ERG response, the ERG amplitude could be expected to be proportional to stimulus area and thus with the square of diameter in which case a slope of two can be expected. The smaller slope suggests the presence of retinal eccentricity dependent changes in cone densities and possibly other factors such as cone to bipolar cell convergence.

For stimuli larger than 70° (i.e. for eccentricities beyond 35°) the high temporal frequency L- and M-cone driven responses had different characteristics: whereas M-cone driven responses with FF and 70° diameter stimuli had similar amplitudes, L-cone driven responses were substantially larger for FF stimuli. This finding suggests that beyond 35° eccentricity, ERG responses are largely generated by L-cone activity. This is in agreement with previous multifocal ERG data [14] and with eccentricity dependent mRNA expression [12,13] suggesting that L-cones are almost exclusively present in the far periphery. However, as discussed in the next paragraph, FF M-cone driven responses were larger than those obtained with other stimulus configurations at 48 Hz, indicating that M-cones are not completely absent in the far periphery.

### Complex signal interactions at high temporal frequencies

Three aspects of the present data show that even within the high frequency range temporal frequency has a complex influence on the ERG responses (Fig. 3). Firstly, the L/M ratios for all but the FF stimuli decreased from about two at 30 Hz to slightly more than one at 48 Hz. Secondly, the difference between FF responses and those at other stimulus configurations increased with increasing temporal frequency: the 30 Hz L-cone driven FF responses were about 1.5 times larger than the 70° responses, whereas at 48 Hz this ratio was about four. M-cone driven FF responses at 30 Hz were slightly smaller than those to 70° stimuli. At 48 Hz, they were more than a factor of two larger (see Fig. 3). These results indicate that cone driven responses in the far periphery may have different temporal properties compared to central responses. In FF responses, the vector summation of these responses may lead to complicated interactions. For instance, if central and peripheral M-cone driven responses are more than 90° apart at 30 Hz than the combined (FF) response may be smaller than the 70° response because of destructive interactions. Possibly, they are less than 90° apart at higher temporal frequencies, so that the interaction is additive at these temporal frequencies. Thirdly, stimulus configuration and temporal frequency have an effect on the phase differences between L- and M-cone driven responses for different stimuli (Fig. 5, right plots). Possibly, the three effects are caused by the same mechanism. To test this proposal more detailed data at additional temporal frequencies and stimulus configurations are needed.

## Conclusion

In conclusion, using triple silent substitution, it is possible to study the spatial properties of L- and M-cone driven ERG signals into ERGs. The data confirm that ERGs reflect the activity of two different post-receptoral pathways.

## Supporting Information

S1 Fig Amplitude results of Experiment 2. Comparison between 12 and 30 Hz ERG amplitudes measured with black (black triangles) and white surround (open circles) with one subject. Upper and middle plots: L- (left plots) and M-cone (right plots) driven ERG amplitudes. Lower plots: L/M ratio estimated from 12 (left plot) and 30 Hz (right plot) response amplitudes. The red dashed lines indicate unity L/M ratios.
(EPS)

S2 Fig Phase results of Experiment 2. Comparison between 12 and 30 Hz ERG phases with black (black triangles) and white surround (open circles) with one subject. Upper and middle plots: L- (left plots) and M-cone (right plots) driven ERG phases. Lower plots: L-M phase differences estimated from 12 (left plot) and 30 Hz (right plot) response phases.
(EPS)

S3 Fig Amplitude results of Experiment 3. Comparison between 12 and 36 Hz averaged ERG amplitudes of measured with white (open circles) and reddish background (pink triangles). Upper and middle plots: L- (left plots) and M-cone (right plots) driven ERG amplitudes. Errors bars indicate standard deviation. Lower plots: L/M ratio estimated from 12 (left plot) and 36 Hz (right plots) amplitude responses. The red dashed lines indicate unity L/M ratios.
(EPS)

S4 Fig Phase results of Experiment 3. Comparison between 12 and 36 Hz averaged ERG phases measured with white (open circles) and reddish background (pink triangles). Upper and middle plots: L- (left plots) and M-cone (right plots) driven ERG phases. Lower plots: L-M phase difference estimated from 12 (left plot) and 36 Hz (right plots) phases.
(EPS)

S1 File Results Experiment 1 subject JK. (XLSX)

S2 File Results Experiment 1 subject KT. (XLSX)

S3 File Results Experiment 1 subject MC. (XLSX)

S4 File Results Experiment 1 subject MJ. (XLSX)

S5 File Results Experiment 2 subject MJ. (XLSX)

S6 File Results Experiment 2 all subjects. (XLS)
